# ENDOCRINOLOGY IN THE TIME OF COVID-19: Management of Cushing's syndromeThis manuscript is part of a commissioned series of urgent clinical guidance documents on the management of endocrine conditions in the time of COVID-19. This clinical guidance document underwent expedited open peer review by Jérôme Bertherat (Université Paris Descartes, Paris, France), André Lacroix (University of Montreal, Canada), Alberto Pereira (Leiden University, The Netherlands), and Peter Trainer (University of Manchester, UK)

**DOI:** 10.1530/EJE-20-0352

**Published:** 2020-07-01

**Authors:** John Newell-Price, Lynnette K Nieman, Martin Reincke, Antoine Tabarin

**Affiliations:** 1 Department of Oncology and Metabolism, Medical School, University of Sheffield, Sheffield, UK; 2 The National Institute of Diabetes and Digestive and Kidney Diseases (NIDDK), National Institutes of Health, Bethesda, Maryland, USA; 3 Department of Medicine IV, Klinikum University of Munich, Munich, Germany; 4 Service d'Endocrinologie – Diabète et Nutrition, CHU de Bordeaux, Bordeaux, France

## Abstract

Clinical evaluation should guide those needing immediate investigation. Strict adherence to COVID-19 protection measures is necessary. Alternative ways of consultations (telephone, video) should be used. Early discussion with regional/national experts about investigation and management of potential and existing patients is strongly encouraged. Patients with moderate or severe clinical features need urgent investigation and management. Patients with active Cushing's syndrome, especially when severe, are immunocompromised and vigorous adherence to the principles of social isolation is recommended. In patients with mild features or in whom a diagnosis is less likely, clinical re-evaluation should be repeated at 3 and 6 months or deferred until the prevalence of SARS-CoV-2 has significantly decreased; however, those individuals should be encouraged to maintain social distancing. Diagnostic pathways may need to be very different from usual recommendations in order to reduce possible exposure to SARS-CoV-2. When extensive differential diagnostic testing and/or surgery is not feasible, it should be deferred and medical treatment should be initiated. Transsphenoidal pituitary surgery should be delayed during high SARS-CoV-2 viral prevalence. Medical management rather than surgery will be the used for most patients, since the short- to mid-term prognosis depends in most cases on hypercortisolism rather than its cause; it should be initiated promptly to minimize the risk of infection in these immunosuppressed patients. The risk/benefit ratio of these recommendations will need re-evaluation every 2–3 months from April 2020 in each country (and possibly local areas) and will depend on the local health care structure and phase of pandemic.

***‘Il meglio e l'inimico del bene’ (or …‘perfection is the enemy of the good’)***

Voltaire, Dictionnaire Philosophique, 1770

## Introduction

The COVID-19 pandemic declared by the WHO in March 2020 requires endocrinologists to adapt to practices that do not sit easily with our norm of using detailed investigation and monitoring to achieve perfection. This is particularly the case for Cushing's syndrome, where management is complex and challenging even in the best of times. Here, we focus on key areas of the patient pathway to give practical advice for clinical practice and highlight where these differ from usual recommendations and guidelines.

## Principles of care

- Minimise outpatient attendance at time of high SARS-CoV-2 virus prevalence to reduce risks of COVID-19 illness for patients and hospital staff. Telephone/video clinics should be used as the preferred option for the vast majority of patients.
- Patients with active Cushing's syndrome are immunosuppressed and at risk of viral and other infections and should be advised to follow their government's guidance on social distancing and self-isolation/shielding, including taking sick leave; rapid normalization of cortisol secretion is needed to minimize the risk of infection.
- Since diabetes mellitus and hypertension appear to be significant risk factors for adverse outcomes from COVID-19, these co-morbidities should be very actively managed.
- Minimise imaging requests at time of high COVID-19 virus prevalence to reduce risks to patients and hospital staff and emergency pressure on the radiology service.
- Surveillance imaging and laboratory investigations in otherwise stable patients should be deferred at time of high SAR-CoV-2 virus prevalence and greater reliance placed on clinical assessment.
- Good communication with patients is essential to explain the potential trade-offs that will result from instituting this guidance with respect to survival, quality of life and functional outcomes and the risk of acquiring COVID-19 and the resulting sequelae.
- Discuss suspected/known Cushing's syndrome patients with recognised experts in Cushing's syndrome in your country to facilitate management. This is especially true where access to diagnostic and therapeutic resources is limited.
- The risk/benefit ratio of these recommendations will need re-evaluation every 2–3 months from April 2020 in each country (and possibly local areas) and will depend on the local health care structure and phase of pandemic and how health care providers are able to structure care for patients with and without COVID-19, with a gradual return to more standard care.

## Diagnosis

Clinical features: At the best of times, diagnosis and differential diagnosis of Cushing's syndrome may be challenging. During the COVID-19 pandemic, the following principles should be applied:

- Attention must be given to the key clinical features that are more discriminating for Cushing's syndrome (1), so as to investigate those for whom the diagnosis is more likely and to avoid or defer investigation where the diagnosis is less likely.
- If clinical features are mild, or in doubt, investigation should be deferred for 3 to 6 months after a repeat clinical assessment and/or until SARS-CoV-2 viral prevalence has significantly diminished. This is justified on the basis that the benefits of treating the cause of Cushing's syndrome in patients with mild disease is not fully established (2).
- Judgement of severity may need to be made by telephone, with assessment of the rapidity of symptom onset; video consultation may allow assessment of physical features.
- When diagnostic testing is deferred, treatment of potential co-morbidities (such as hypertension and diabetes) should be optimized.
- Those with moderate and severe clinical disease must be investigated and managed urgently, since these patients are prone to develop various co-morbidities requiring hospitalization and/or have immunosuppression that may potentially facilitate viral or other infections.
- To facilitate triage, when referring patients to centres, the details of co-morbidities (BP, glucose etc.) as well as any known relevant biochemistry (e.g. serum cortisol) should be included.
- Patients with adrenal incidentaloma should only be investigated for hypercortisolism at times of high SARS-CoV-2 viral prevalence if radiological features suggest adrenocortical cancer or clinical signs suggest moderate to severe Cushing's syndrome (3).

### Biochemical investigations

The following principles are suggested:

- Overnight dexamethasone suppression testing and 24-h urinary free cortisol are recommended as first line tests, with measurement of serum electrolytes and glucose, complete blood count, CRP and HbA1c.
- A basal plasma sample for ACTH should be measured at the beginning of the investigation process to allow rapid stratification into ACTH-dependent and independent disease, together with basal anterior pituitary function and androgens.
- Salivary cortisol/cortisone tests should be avoided due to the potential for viral contamination and infection of laboratory staff, unless the laboratory has instituted appropriate and approved measures to handle biohazard material.
- In the context of severe clinical signs of Cushing's syndrome and in the absence of major stressors (e.g. sepsis), a single measurement of serum cortisol may be sufficient to confirm the diagnosis if very elevated – for example, >1000 nmol/L (37 µg/dL), especially in the presence of an elevated neutrophil count and hypokalaemia (4). It is important to remember that oral oestrogens may cause higher measured serum cortisol due to increased CBG. Similarly, a single UFC >5× ULN is highly suggestive of Cushing's syndrome.

## Differential diagnosis

**Figure 1:**
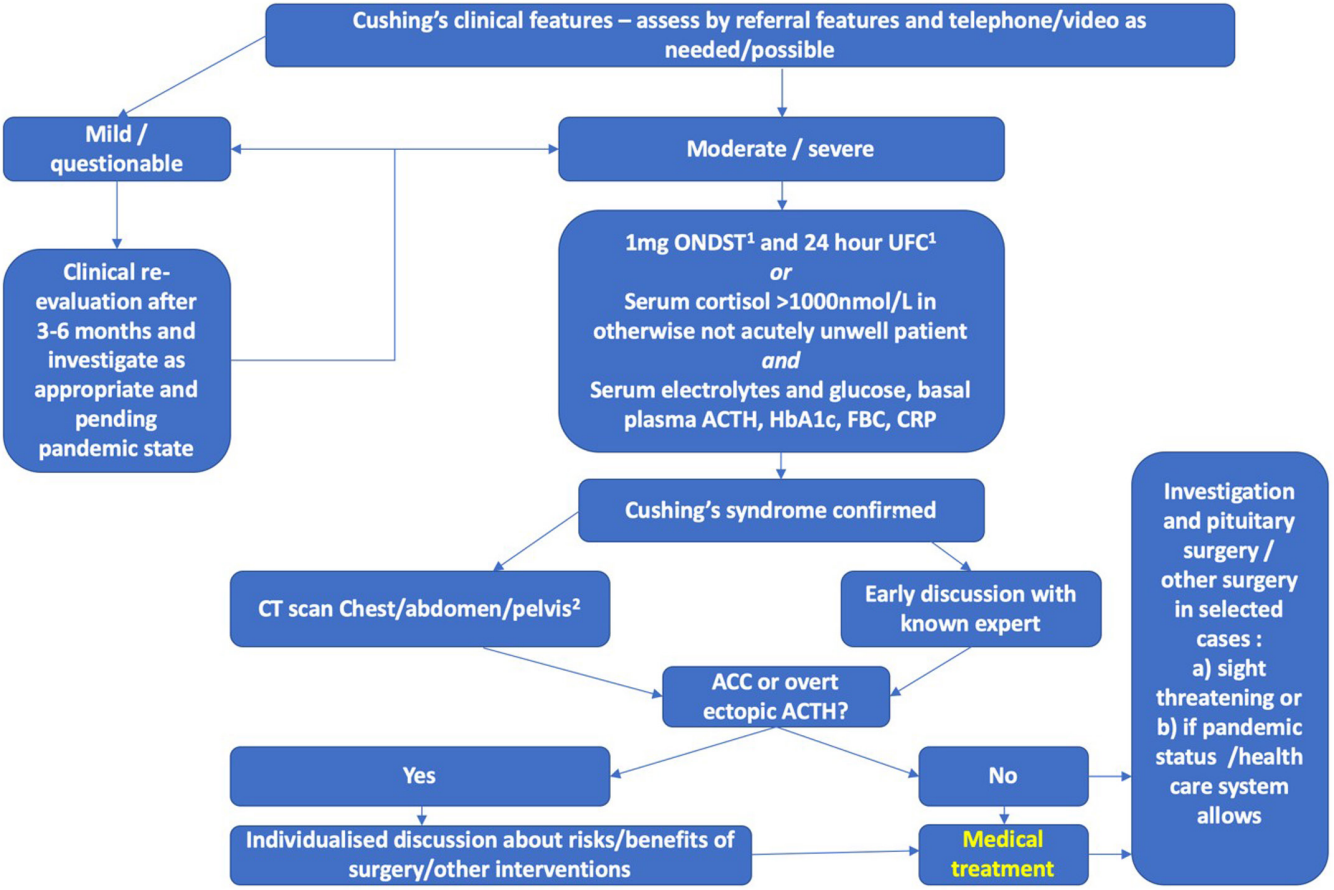
Suggested algorithm for investigation and management of suspected Cushing's syndrome during COVID-19 pandemic. ONDST: overnight dexamethasone suppression test; UFC: urinary free cortisol; ACC: adrenocortical cancer. ^1^The higher the UFC or post-dexamethasone serum cortisol the greater the confidence that the patient has Cushing's syndrome; ^2^MRI or CT pituitary if clear visual field defects to identify sight-threatening macroadenoma – CT head with coronal reconstructions can be included in the body CT to minimise need for extra imaging.

The usual detailed strategies for investigation of the cause of Cushing's syndrome have to be modified significantly during the COVID-19 pandemic. The following recommendations depart significantly from standard guidelines (Fig. 1):

- Once Cushing's syndrome is confirmed or highly likely, patients should undergo an immediate CT scan of thorax (with 1 mm slice thickness), abdomen and pelvis (with 2–5 mm slice thickness) to identify adrenocortical cancer and overt disease causing the ectopic ACTH syndrome. CT will immediately identify significant disease that may need urgent cancer surgery (e.g. adrenal cancer) or other investigation and management such as for small cell lung cancer. Also, it allows identification of major co-morbidities (vertebral fractures, infectious foci, pulmonary thromboembolism and atherosclerosis), which may guide further clinical care.
- Cushing's disease is the most frequent aetiology of Cushing's syndrome, and the combination of clinical factors including reproductive age, female sex, slow onset of symptoms over several years, moderate increase in UFC (<4× ULN) and ACTH (plasma <100 pg/mL) have a high predictive value of Cushing's disease.
- If there is visual field compromise on clinical examination and or severe headaches, pituitary imaging by MRI or CT should be performed. It should be noted, however, that a corticotroph macroadenoma is an unusual cause of moderate/severe Cushing's syndrome.
- If there is no visual compromise, it is reasonable to not perform a pituitary MRI, as in most cases once the diagnosis of Cushing's syndrome is confirmed, management should be with medical treatment, for at least 3 to 6 months, and while the prevalence of SARS-CoV-2 remains high. Furthermore, the confined nature of MRI scanners represents a potential transmission vector, especially in localities where patients with COVID-19 are being treated. Nevertheless, these suggestions may need modifying to more normal standards of care depending on the state of the local health care system and pandemic at that location.
- All other investigations should usually be avoided at the time of high SARS-CoV-2 viral prevalence as they will not result in specific management because:
  - Transsphenoidal pituitary surgery should be avoided: this procedure results in aerosol formation which conveys a very high risk of viral transmission (5); it remains an option in urgent patients (or for other patients in the chronic phase of the pandemic) after negative SARS-CoV-2 testing if hospital resources are available for safe care and the operation will be performed by a highly experienced pituitary surgeon.
  - Other testing including peripheral CRH or desmopressin testing or bilateral petrosal sinus sampling can be deferred until SARS-CoV-2 prevalence has decreased.
  - Once COVID-19 prevalence has decreased in a given society/locality, consideration can be given to stopping the medical treatment and initiating additional investigation of the aetiology once the patient is again hypercortisolaemic. Such an approach is justified on the basis of the key principles of care mentioned previously and the fact that some centres already routinely consider medical pre-treatment ahead of surgery (2). In addition, other than causes that specifically affect prognosis (ACC, pituitary macroadenomas, overtly resectable ectopic ACTH-secreting tumours), the short- to mid-term prognosis of Cushing's syndrome is related to the cortisol excess and not its cause.

## Treatment

### Overview

During high SARS-CoV-2 viral prevalence, surgery for Cushing's syndrome should be avoided or altered in most health care settings because:

- The risks to patients of contracting COVID-19, increasing immune suppression from major surgical intervention and the potential risk to health care workers (e.g. transsphenoidal surgery as mentioned previously).
- If benefits of transsphenoidal surgery outweigh the risks (e.g. very active disease, difficult to control hypercortisolism despite steroidogenesis inhibitors, side effects of medical treatment), surgery should be done using appropriate protection (face masks with the highest level of protection for medical procedures – e.g. FFP3 masks) after repeat negative testing for COVID-19 and only by a highly experienced pituitary surgeon.
- When surgical intervention for Cushing's disease is needed because of visual compromise due to optic chiasm compression by a pituitary macroadenoma, consideration may also be given to a limited ‘eyebrow’ craniotomy to avoid the formation of aerosol droplets that occurs during transsphenoidal surgery (see previous section (5)).
- Surgery can be deferred in the context of an imaging lesion highly suggestive of well-differentiated neuroendocrine tumour as an ectopic source of ACTH (e.g a pancreatic or lung lesion), if delay for up to around 3–6 months is unlikely to reduce life expectancy. The mainstay of treatment for patients is medical therapy to control hypercortisolaemia and its effects. If there is delay to surgery, empirical use of somatostatin analog therapy (in addition to steroidogenesis inhibitors) can be considered for their anti-tumour effects in patients with tumours that are, or are highly likely to be, well-differentiated neuroendocrine tumours, even without performing somatostatin receptor imaging.
- Any patients diagnosed with a cancer causing Cushing's syndrome will need an individualised discussion of the risks vs benefits of cancer therapy during COVID-19, either with surgery and/or medical approaches (6).
- Surgery should be considered only in patients who cannot be controlled with medical therapy or those requiring biopsy or resection because of possible cancer.
- When there is lower SARS-CoV-2 viral prevalence and when appropriate institutional measures have been implemented to provide safety for patients, elective surgical transsphenoidal surgery and other surgical procedures can be reconsidered. It is implicit that the appropriate diagnostic procedures will be needed prior to these.

### Medical therapy

The following principles are recommended:

- Co-morbidities should be treated as for standard of care (2).
- Avoid initiation of ACE inhibitors or AT1 receptor blockers for treatment of hypertension until their influence on susceptibility to SARS-CoV-19 infection is clarified (7), but it is not suggested to stop these in those patients already on treatment with satisfactory control.
- For most patients, steroidogenesis inhibitors will be the mainstay of treatment since they treat all types of Cushing's syndrome.
- A glucocorticoid receptor (GR) antagonist also may be used but is difficult to titrate and is not universally available (8) and is only indicated for treatment of hypertension and diabetes mellitus in Cushing's syndrome.
- Patients with severe Cushing's syndrome should receive prophylaxis for *Pneumocystis jivoreci* with trimethoprim/sulfamethoxazole (2). In patients with cough, fever and respiratory distress, differentiation should be made between COVID-19 infection and other pulmonary infections, such as *Pneumocystis jivoreci*, that may share similar CT features, to ensure appropriate treatment.
- It is recommended that treatment with low molecular weight heparin be given until definitive treatment has been achieved, especially in patients with moderate to severe disease (2).
- Dopamine agonists and somatostatin analogs do not reliably lower plasma ACTH and serum cortisol in all patients and are not recommended as mono-therapy in a patient needing urgent biochemical control.
- Regular telephone (or, if possible, video) consultations are recommended to assess response to treatment, including symptoms of hypoadrenalism and self-reported weight, BP and capillary glucose.

### Monitoring and initiation of medical therapy

#### Principles

**Figure 2:**
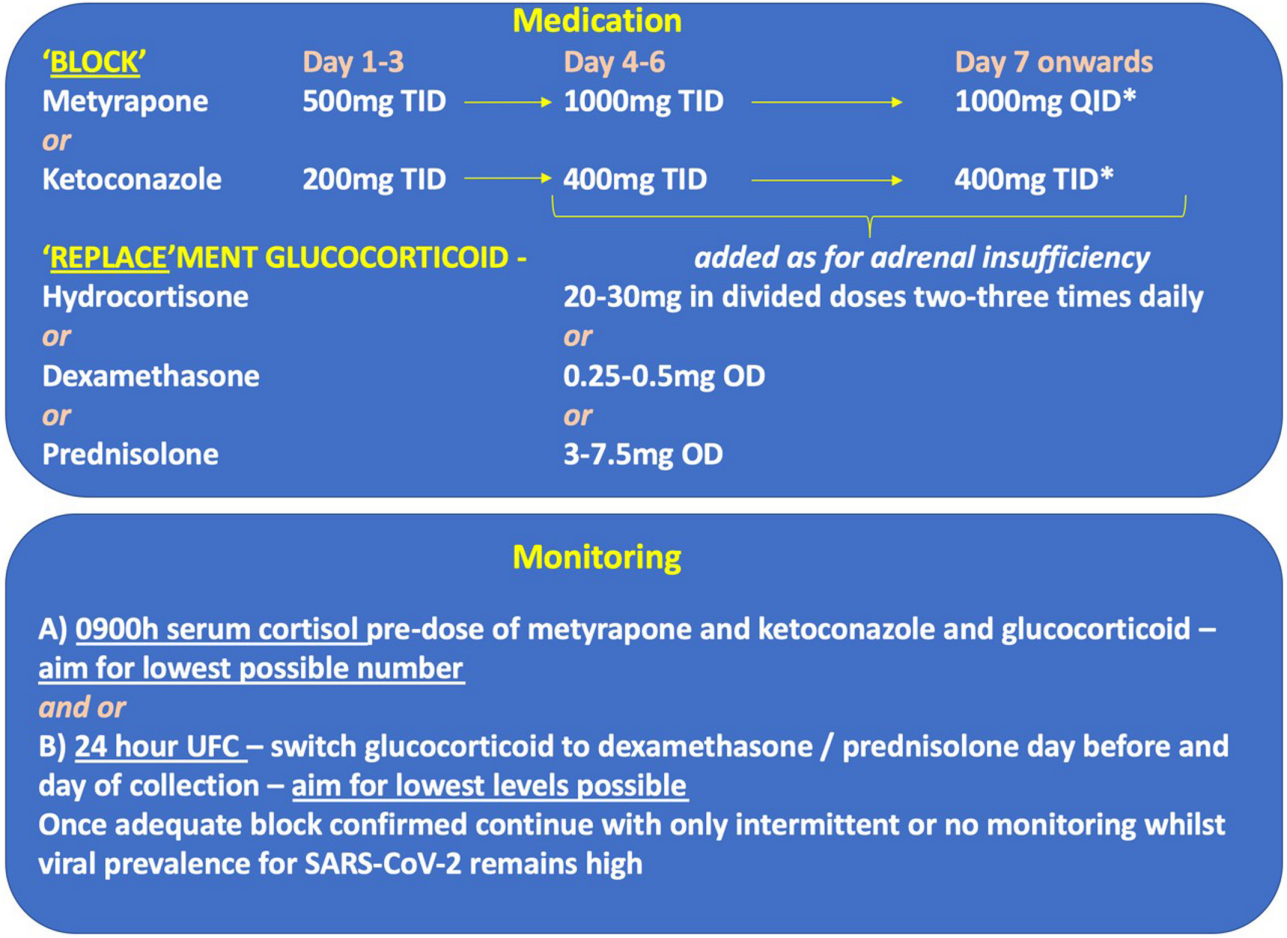
Medical management by ‘Block and Replace’. These recommendations for block and replace regime are ‘expert advice’ rather than being evidence based. Telephone consultations should evaluate symptomatology, weight, BP and capillary blood glucose to assist judging the effectiveness of the replacement glucocorticoid dose. Patients should be instructed to follow ‘sick day rules’ for glucocorticoid replacement (see text). Note: when using metyrapone any assay that has cross-reactivity with 11-deoxycortisol will read falsely high – where possible – LC-MS/MS assays are recommended; *doses may be increased further if needed and ketoconazole and metyrapone may be used in combination; with monitoring, it may also be possible to reduce the dose while maintaining blockade – collaboration with expert in Cushing's is recommended.

- Where possible, it is suggested that the majority of patients, especially those with more severe clinical features or any features that suggest cyclical disease, are considered for a ‘block and replace’ approach when steroidogenesis inhibitors are used (Fig. 2). Once established, this approach limits the need for biochemical monitoring and reduces the risk of adrenal insufficiency. The suggested regime is for a full ‘block and replace’, but this may be modified in collaboration with experts in Cushing's to a partial ‘block’ regime with regular telephone monitoring of symptoms.
- Urinary free cortisol (UFC) measurement allows remote monitoring for all patients (collect urine for 24 h, measure volume, post aliquot to lab) except for those on GR antagonists and those on block and replace regimes that use hydrocortisone. UFC measurement cannot be used to identify overtreatment on a dose titration regime. If using metyrapone, a mass spectrometry assay is needed to measure UFC as metyrapone inhibits CYP11B1, resulting in accumulation of tetrahydro-11-deoxycortisol, which will cross react with many cortisol immunoassays.
- If dose titration is used, pre-dose serum cortisol values also may be used to estimate control and can be done at a local lab for patients remote from the centre. Assuming a relative lack of diurnal variation, morning cortisol goal values are 250–330 nmol/L (9–12 µg/dL), but higher levels (250–500 nmol/L) may be acceptable in the short-to-medium term if regular monitoring is challenging in order to avoid hypoadrenalism. When using metyrapone, the immunoassay must not cross react with 11-deoxycortisol and, where available, mass spectrometry assays are recommended.

#### Patients on existing long-term treatment

- If the recent clinical state, drug dose and biochemical monitoring for cortisol excess are stable and controlled, patients should be maintained on their current regimen.
- Patients on any form of treatment for Cushing's syndrome can be considered for switching to a block and replace regime (see subsequent section) if that will allow either better control or ease of monitoring with fewer patient visits.
- Consideration should be given to extending the intervals between biochemical monitoring in line with ‘principles of care’ mentioned previously.
- Patients should have access to stress doses of glucocorticoid tablets (e.g. 20 mg hydrocortisone four times daily for up to 2 weeks) and preferably an emergency injection kit for i.m. injection of 100 mg hydrocortisone in case of intercurrent infection or trauma (see https://www.endocrinology.org/clinical-practice/covid-19-resources-for-managing-endocrine-conditions/)
- Patients need education about ‘sick day rules’ and to take extra glucocorticoid if unwell and to liaise with their treating physician/nurse specialist as needed via a designated contact telephone/email or other means. It is best for patients to have written instructions for stress dose adjustments and other emergencies.

#### Patients needing initiation of treatment

- Country-specific availability will determine which agent can be used. Patients on ketoconazole, however, should have liver function monitored every month (deviating from EMA guidance for weekly testing) for the first 3 months at the start of treatment or following a dose increase as this has been found to be safe (9). Ketoconazole also has a slower onset of action than metyrapone and has a greater number of drug-drug interactions because of inhibition of CYP3A4. For these reasons, where available, metyrapone is suggested as being preferable.
- Ketoconazole needs an acid gastric environment to be absorbed (avoid proton pump inhibitors).
- Hypokalaemia may be exacerbated by metyrapone and GR antagonists; this should be anticipated and treated with potassium supplements or mineralocorticoid antagonists.
- Some biochemical monitoring will likely be required for any of these agents.
- Combination treatment with ketoconazole and metyrapone can be considered from the outset in severe cases (10) and requires monitoring of liver biochemistry as mentioned previously.
- If a patient on medical treatment for Cushing's syndrome is infected by SARS-C0V-2, it is recommended that stress doses of glucocorticoid are given.

## Disclaimer

Due to the emerging nature of the COVID-19 crisis, this document is not based on extensive systematic review or meta-analysis but on rapid expert consensus. The document should be considered as guidance only; it is not intended to determine an absolute standard of medical care. Healthcare staff need to consider individual circumstances when devising the management plan for a specific patient.

## Declaration of interest

The authors declare the following interests: JN-P, AT and MR have worked as consultants and/or received speaker fees from HRA Pharma, Novartis, and Recordati. LN has no disclosures.

## Funding

This guidance did not receive any specific grant from any funding agency in the public, commercial or not-for-profit sector.
